# White Grape Skin Extraction, Analytical Profile, and Biological Activity: From the Laboratory to the Industrial Scale Within a Circular Economy Framework

**DOI:** 10.3390/ph18091373

**Published:** 2025-09-13

**Authors:** Larissa Della Vedova, Giovanna Baron, Paolo Morazzoni, Sandro Santinello, Safwa Moheb El Haddad, Jose Antonio Valdés-González, Stefano Piazza, Mario Dell’Agli, Giancarlo Aldini, Francesca Gado

**Affiliations:** 1 Department of Pharmaceutical Sciences, University of Milan, Via Mangiagalli 25, 20133 Milan, Italy; l.dellavedova@uu.nl (L.D.V.); giovanna.baron@unimi.it (G.B.); giancarlo.aldini@unimi.it (G.A.); 2 Distillerie Bonollo Umberto S.p.A., Nutraceutical Division, Mestrino, 35035 Padova, Italy; paolo.morazzoni@bonollo.it (P.M.); sandro.santinello@bonollo.it (S.S.); 3 Department of Pharmacological and Biomolecular Sciences, University of Milan, 20133 Milan, Italy; safwa.elhaddad@unimi.it (S.M.E.H.); stefano.piazza@unimi.it (S.P.); mario.dellagli@unimi.it (M.D.); 4 Departamento de Farmacología, Farmacognosia y Botánica, Facultad de Farmacia, Universidad Complutense de Madrid, Plaza Ramón y Cajal s/n, Ciudad Universitaria, 28040 Madrid, Spain; joseaval@ucm.es

**Keywords:** grape by-products, polyphenols, antioxidant, anti-inflammatory, LC-HRMS, metabolomics

## Abstract

**Background:** The sustainable use of agro-industrial by-products is essential to reduce environmental impact and enhance resource efficiency. In this study, white grape skins (WGSs), a distillation by-product of grappa production, are valorized through the development of an eco-friendly extraction process. **Methods:** At the laboratory scale, water-based and hydroalcoholic extractions are evaluated, prioritizing the water-based method due to its better scalability and eco-sustainability. Furthermore, this green extraction method enables industrial scale-up by Distillerie Bonollo Umberto S.p.A. (Mestrino, Italy), resulting in Vituva^®^, an industrial extract with a composition comparable to its water-based laboratory counterpart. LC-HRMS-based targeted metabolomics identified 50 metabolites in the hydroalcoholic extract, 36 in the water-based extract, and 37 in the industrial extract, which included mainly polyphenols such as flavonoids and phenolic acids. **Results:** In vitro assays show that the water-based and industrial extracts exhibit significant anti-inflammatory activity, especially in gastric epithelial cells, while the hydroalcoholic extract displays stronger antioxidant activity via Nrf2 pathway activation but was more cytotoxic, possibly due to polyphenol-induced hormesis. Notably, the industrial extract also activates Nrf2 to a lesser extent, supporting its dual bioactivity profile. Chemoinformatic and statistical analyses support the identification of the likely mechanisms of action. **Conclusions:** Overall, this work demonstrates how green chemistry and circular economy principles transform a waste product into a high-value bioactive ingredient.

## 1. Introduction

The growing environmental pressure caused by the extraction of natural resources and waste generation underscores the urgent need for sustainable solutions. The circular economy offers a promising framework to optimize resource use and reduce environmental degradation.

However, to ensure truly sustainable outcomes, it is crucial to critically assess and align circular economy practices with ecological and societal values [1]. Briefly, the concept of a circular economy contrasts with the linear economy, which is characterized by the conversion of natural resources into waste through production, resulting in environmental degradation and polluting ecosystems. A circular economy aims to minimize environmental impact by restoring ecological damage from resource extraction and reducing waste generation throughout a product’s lifecycle, emphasizing biogeochemical cycle integration and product recycling to ensure a closed-loop system with minimal resource loss [2]. In the context of the wine and grappa production supply chain, the principles of a circular economy can be particularly impactful. The traditional linear model generates large amounts of waste, including grape pomace and other by-products, whereas circular economy practices allow their repurposing, reducing environmental impact, and creating additional value. Wine production is globally significant, with Italy as the leading producer, accounting for 19.3% of the global output and producing 50.2 million hectoliters in 2021 [3]. However, this large-scale production also generates a substantial amount of by-products and waste, such as grape pomace, stems, and seeds, making waste management and resource reutilization critical to reducing the environmental impact of winemaking. In this context, circular economy principles offer a promising solution by transforming these underexploited residues into high-value materials, thereby reducing waste and supporting sustainable development [4]. Grape pomace alone, produced at about 23 million tons annually in Europe, contains valuable compounds such as grape seed oil and polyphenols that can be recovered and reused [5,6]. By minimizing transport time and adopting strategies such as the “Universal Recovery Strategy,” the wine industry can close the loop, turning waste into resources. This both reduces environmental impact and adds economic value, fostering a more sustainable production model [7].

Worldwide, the agri-food sector faces similar challenges, generating large amounts of by-products that are often discarded despite being rich in bioactive compounds. Harnessing this potential is crucial to meet both environmental and economic goals. Conventional extraction methods based on organic solvents have been widely used but suffer from drawbacks such as toxicity, volatility, and non-renewability [8]. In contrast, “green extraction” techniques have emerged as sustainable alternatives, enabling efficient recovery with eco-friendly solvents, lower energy consumption, and greater preservation of bioactives [9,10]. These include ultrasonic, microwave, pulsed electric fields, pressurized hot water, and subcritical fluid extraction. Their application not only reduces vinification residues and disposal costs but also generates additional income for producers, while providing industries and consumers with high-value bioactive compounds [10,11].

This work describes a study conducted in collaboration with Distillerie Bonollo Umberto S.p.A. (Mestrino, Italy), which provided white grape skins (WGSs), a distillation residue produced in several tons per day. Given this large amount, it is essential to valorize and reclassify it in a “co-product”, reducing its environmental burden, as well as generating high added-value materials [12]. WGSs are a valuable source of polyphenols and have potential applications as nutraceuticals, since grape skins are widely reported to contain bioactive compounds with beneficial effects in models of chronic diseases [13,14,15]. The aim of this study is to develop and scale up, in collaboration with the company, a green extraction method using only water as a solvent, chosen for its environmental impact, reproducibility, and cost-effectiveness. In this study, water-based extracts (WEs, laboratory scale) and industrial-scale water extracts (IEs) are compared with hydroalcoholic extract (HE, ethanol: water 70:30). Both WE and IE show a rich analytical profile and, importantly, exhibit greater safety in cell viability assays compared to HE. Their antioxidant and anti-inflammatory activities are further assessed in cellular models using gene reporters for the transcription factors Nrf2 (nuclear factor erythroid 2-related factor 2) and NF-κB (nuclear factor-kappa B), as well as in a human gastric cell line (GES-1). Previous work already validated the anti-inflammatory effects of grape-derived extracts at the gastric level, thus reinforcing the potential valorization of WGSs for nutraceutical purposes [16].

Taken together, the valorization of WGSs through green extraction and in vitro biological assays establishes this distillation residue as a sustainable and promising source of bioactive compounds with potential nutraceutical applications.

## 2. Results and Discussion

### 2.1. Extraction Yield

The yield of extraction of dried white grape skins (WGSs) with the three different techniques, as described in Section 3.2, is reported in Table 1.

Compared to the WE and IE, the HE exhibits a lower extraction yield, while the yields of the WE and IE are within the same order of magnitude.

Water-based extractions result in a higher yield compared to the hydroalcoholic method. This is because the aqueous methodology involves a single step, which facilitates the extraction of hydrophilic compounds, including certain subclasses of polyphenols such as flavonols, flavanols, and phenolic acids. The hydroalcoholic method undergoes a specific enrichment in polyphenols through two main steps: the first is the actual extraction under magnetic stirring, and the second involves polyphenol enrichment on a specific resin (Sepabeads SP207, Thermo Fisher Scientific, San Jose, CA, USA), thus removing all the non-polyphenol compounds. From a green chemistry and scalability perspective, the production of this hydroalcoholic extract is not considered feasible.

Ethanol is regarded as a green solvent, especially when used in a mixture with water [17], but it poses hazard issues for industrial-scale use since the exposure to high concentrations of ethanol vapors may cause eyes, skin, and respiratory tract irritation, as well as narcosis, sleepiness, impaired perception, and lack of coordination [18], while water is considered the most environmentally friendly solvent and poses no risks for industrial use [19]. As a consequence of these considerations, the industrial facility at Distillerie Bonollo Umberto S.p.A. (Mestrino, Italy) considers only WE eligible for an industrial scale-up and thus performs a water polyphenol extraction from grape skins as described in the Section 3, obtaining a new extract here named IE.

The characterization of the phytochemical profile by HPLC-HRMS of WE and IE is carried out, as well as for HE, since this represents the standard polyphenol extraction procedure [20], and therefore, this sample is employed to obtain a comprehensive polyphenol-enriched extract that is crucial for understanding the total metabolite composition of dried white grape skins and for comparison with WE and IE metabolomic profiles.

### 2.2. Analytical Characterization

#### 2.2.1. Untargeted Metabolomics Analysis

The spectral data for untargeted metabolomics from the three extracts were collected using the analytical platform detailed in Section 3.3, operating in both positive and negative electrospray ionization modes (ESI +/−). A data-dependent acquisition (DDA) approach was employed to automatically capture MS2 fragmentation spectra for all precursor ions that exceeded a predetermined intensity threshold. 

**Figure 1:**
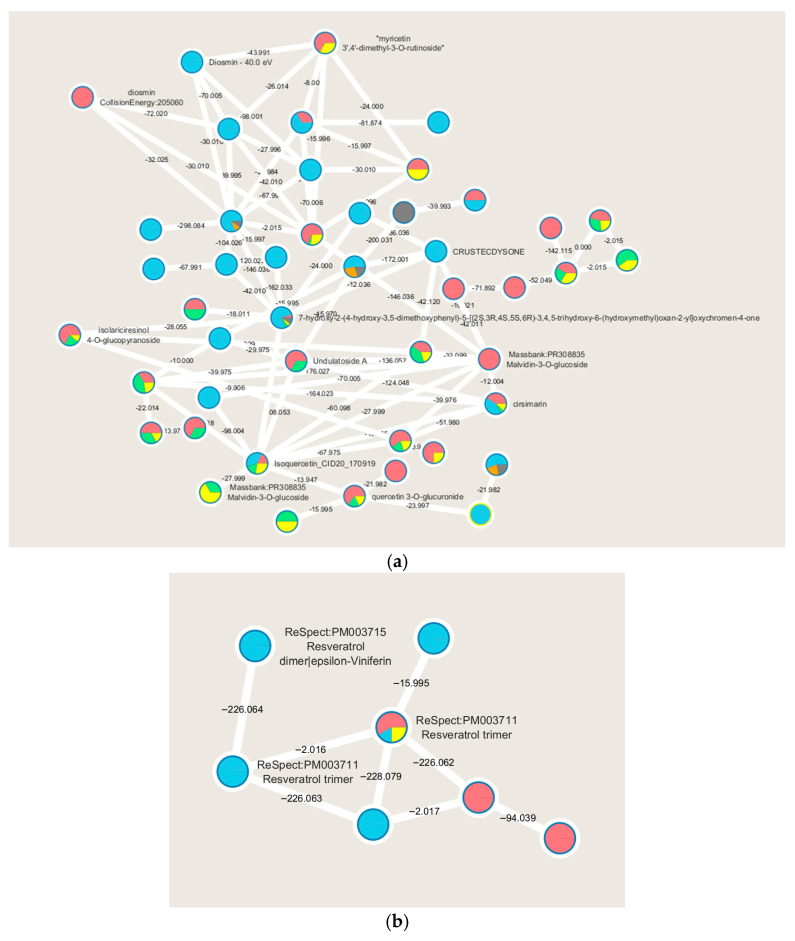
Main molecular families are identified performing the molecular networking of WGS extracts analyzed by LC-MS/MS: flavonoids (**a**), and stilbenes (**b**). Node colors indicate the WGS extracts (red for HE in negative ion mode, light blue for HE in positive ion mode, green for WE in negative ion mode, orange for WE in positive ion mode, yellow for IE in negative ion mode and in gray for IE in positive ion mode) and the respective MS2 spectral counts, which indicate the presence or absence of metabolites. ReSpect refers to the reference spectrum.

To gain a clearer understanding of the diverse metabolites present in the extracts, which vary in polarity and distribution across the whole WGS starting material, a visual examination of the chromatographic profiles was conducted. This step aims to highlight the broad spectrum of metabolites and their differential patterns. For a deeper exploration of the chemical classes and metabolomic profiles associated with WGS, the acquired spectral data are analyzed using molecular networking on the Global Natural Products Social Molecular Networking (GNPS) platform. This approach provides a comprehensive overview of molecular insights derived from MS/MS data. The GNPS algorithm assesses similarities between spectra both within and across samples, organizing ions within a specified mass tolerance into consensus spectra, which are visualized as nodes. Metabolites with structural similarities and comparable gas-phase chemistries were grouped into molecular families, based on cosine similarity scores of 0.7 or higher [21]. The free software Cytoscape 3.10.3 is used for the representation of molecular networks [22]. In the resulting molecular network (Supplementary Data, Figure S1), 1212 consensus spectra (nodes) are identified, forming 118 distinct molecular families (each comprising at least two interconnected nodes). Spectra that do not cluster into molecular families are displayed as self-loop nodes at the network’s base. Within this network (Figure 1), 19 nodes were tentatively annotated using automated spectral matching against reference libraries. These annotations provide preliminary insights into the chemical composition of WGSs, while also underscoring the intricate complexity of the *Vitis vinifera* L. metabolome.

A significant number of compounds remain unknown, while molecular networking highlights other compounds of interest belonging to the polyphenols molecular class, more specifically flavonoids, as evidenced by the molecular family cluster where several of them are putatively annotated (Figure 1a). Flavonoids are widely distributed in fruits and vegetables and are described as having numerous health-promoting effects, including antioxidant, anti-proliferative, and anti-bacterial activities. In plants, flavonoids play key roles in growth and in protection against oxidative stress, including that caused by UV-A and UV-B [23].

WGSs are highlighted as rich in flavonoids, which are polyphenols biosynthesized through the phenylpropanoid pathway. Specifically, these putatively identified metabolites are related to the flavanol biosynthesis pathway. The recognition of this metabolic pathway is significant as it indicates the quality of the raw material used to produce these extracts. In white grapes, flavonoids play a crucial role in conferring flavor to the final product and are directly correlated with specific grape cultivation techniques [24]. Of particular interest is the molecular cluster of resveratrol, present in monomeric, dimeric, and trimeric forms (Figure 1b). Resveratrol is a stilbenoid known to be present in various grape varieties and recognized for its diverse biological activities, such as anti-proliferative, antiviral, neuroprotective, anti-inflammatory, and anti-aging effects [25].

#### 2.2.2. Targeted HPLC-HRMS Analysis for Polyphenol Identification

Table 2 lists all metabolites belonging to the molecular class of polyphenols that are putatively identified by a targeted approach in all three samples and in both polarities. The HE sample is confirmed to be the extract with the highest phytochemical complexity, both in numerical terms (50 identified metabolites) and in the greater variety of metabolites, including anthocyanidins. The WE sample shows lower complexity (36 identified metabolites) and predominantly contains molecules belonging to the class of phenolic acids, as reported in the literature in aqueous extracts. The IE sample, on the other hand, displays a very interesting phytochemical profile, as it contains characteristic molecules of its patented extraction methodology, such as procyanidins. This sample lies midway between the two previously described extraction methods; specifically, it includes 37 identified metabolites predominantly belonging to both the class of phenolic acids, and is characterized by the presence of tartaric acid, which represents the major organic component in grapes and their derivatives [26].

**Figure 2:**
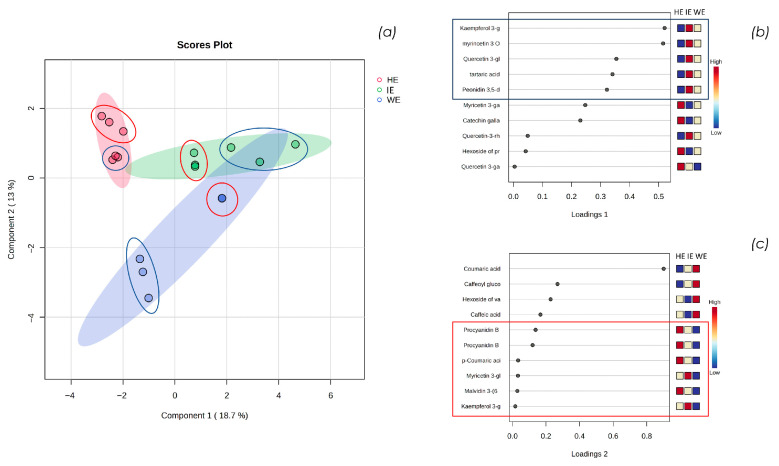
(**a**) shows the sparse-Partial Least Squares Discriminant Analysis (s-PLSDA) 2D score plot of HE, WE, and IE samples performed with MetaboAnalyst 6.0; (**b**) shows the loading plot of component 1, and (**c**) shows the loading plot of component 2.

To further explore and visualize the distribution of metabolites across the three samples, Figure 2 shows the 2D score plot (Figure 2a) and loading plots (Figure 2b,c) for each component obtained from the statistical analysis carried out with MetaboAnalyst 6.0. These plots provide a comprehensive comparison of the metabolite profiles, allowing a focus on the differences in metabolites extracted using the various methods previously discussed. The analysis of these plots facilitates the identification of both shared and unique metabolites associated with each extraction technique, offering a deeper understanding of their distinct biochemical characteristics.

Statistical analysis conducted with Metaboanalyst 6.0 confirms the initial observations: the IE sample partially overlaps with the dimensional areas characterizing HE and WE, indicating that, from a qualitative and semi-quantitative perspective, the industrially finalized extract represents an intermediate between the extract with a more varied polyphenolic profile (HE) and the one predominantly containing phenolic acids (WE). Specifically, in Figure 2a, the triplicates of the semi-quantitative LC-HRMS analyses in positive and negative ion modes are circled in red and blue, respectively. Notably, the variability among replicates in positive ion mode is lower, especially for the WE sample. This is imposable to the reduced number of metabolites identified in this ion mode, primarily anthocyanins. In contrast, the variability among replicates in negative ion mode is greater, both by a higher number of metabolites identified and the greater difficulty in achieving stable ESI ionization [24].

Observing the loading plot of component 1, a similar trend between the WE and IE samples emerges. Specifically, as shown in Figure 2b, the metabolites kaempferol 3-glucuronide, myricetin 3-glucoside/galactoside, quercetin 3-glucoside/galactoside, tartaric acid, and peonidin 3,5-diglucoside are characteristic of component 1. These metabolites are abundant in the IE, significant in the WE, and scarce in the HE.

From the analysis of the loading plot of component 2, a similarity between IE and HE emerges: as shown in Figure 2c, the metabolites procyanidin B, *p*-coumaric acid, myricetin glucoside/galactoside, malvidin 3-(6-coumaroyl)glucoside, and kaempferol 3-glucuronide are abundant in the HE, significant in the IE, and scarce in the WE. This indicates that the hydroalcoholic extraction and the tangential flow extraction facility used in industrial scale-up have a significant impact on the extraction of less polar compounds, such as anthocyanin compounds, compared to the aqueous method in the laboratory scale. Additionally, the IE extract is characterized by the metabolite kaempferol 3-glucuronide, which appears to be a key compound influencing both components. Although this metabolite is detected only in IE, it occurs in trace amounts.

The semi-quantitative LC-HRMS analysis shows that the HE sample is characterized by a significant abundance of malvidin coumaroyl glucoside and malvidin glucoside, which are the two most abundant compounds. This finding reflects the sample preparation, both from the extraction method and the resin enrichment in polyphenols. Regarding the WE sample, the most abundant metabolites are the two coumaric acid glucosides, while for IE, they are tartaric acid and malvidin coumaroyl glucoside. To better evaluate the applications of the industrially finalized IE extract, subsequent in vitro assays were performed on both cellular and non-cellular models.

### 2.3. Total Polyphenol, Anthocyanin, and Tannin Content

Table 3 reports the results obtained with quantitative methods described in Section 3.6 for total polyphenol, anthocyanin, and tannin total contents.

The evaluation of the total polyphenol content is performed using two different methods: high-performance liquid chromatography coupled with a UV-diode array detector (HPLC-UV/DAD) and the Folin–Ciocalteu assay. This choice is justified by the fact that the Folin–Ciocalteu method has a structure–activity relation and limitations [27,28]. The sample HE exhibits the highest total polyphenol content, with the value obtained using the Folin–Ciocalteu method at 51.612% and the more accurate HPLC-UV method value at 43.682%, confirming the effectiveness of enrichment achieved with the Sepabeads SP207 resin. The WE and IE samples show a comparable total polyphenol content, ranging between 2.5% and 3%. The more accurate quantification of the IE sample was obtained with the HPLC-UV method. It is notable that evaluating the total polyphenol content with two different methodologies highlights chemical differences in the IE sample when compared to the WE sample, which shows a superimposable result in the two methods. The Folin–Ciocalteu method provides a structure and antioxidant capacity-related result, as the assay itself is based on a redox reaction; hence, the quantification of polyphenols is influenced by the degree of hydroxylation, presence of allyl carbonilic acids, methoxy groups, flavonols, and flavanones, as these polyphenol subclasses have different reducing power [29]. Nonetheless, this method is useful when the HPLC-UV methodology is unsuitable, as in the case of the HE samples.

In terms of total anthocyanin and tannin content, the HE sample also proves to be the one with the highest content, respectively, at 1.232% and 13.637%. The WE and IE samples, on the other hand, exhibit comparable total tannin content, as reported in Table 3, while the anthocyanins are not detectable in WE. In contrast, anthocyanins are detected in IE and HE, at 0.141% and 1.232%, respectively, indicating that the water extraction is not suitable for the recovery of these phytochemicals.

### 2.4. In Vitro Cellular Viability Assay

**Figure 3:**
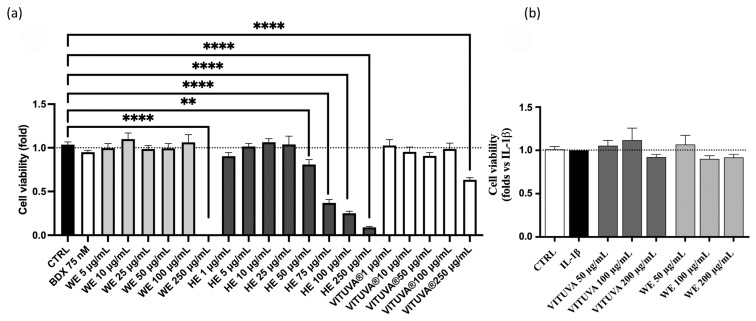
Cell viability of the extracts, measured by MTT assay in HEK293T (**a**) and GES-1 (**b**). Data are expressed as mean ± S.E.M. Statistical analysis was calculated by one-way ANOVA with Bonferroni’s multiple comparison test (** *p* < 0.01, **** *p* < 0.0001 vs. CTRL).

An MTT (3-(4,5-dimethylthiazol-2-yl)-2,5-diphenyltetrazolium bromide) assay was performed on the three extracts to evaluate the viability of the Human Embryonic Kidney 293 (HEK293) cell line treated with increasing concentrations of WE, HE, and IE. WE and IE were assessed to be safe up to 100 µg/mL, whereas HE shows toxicity starting at 50 µg/mL. For this reason, HE is excluded from subsequent experiments, while WE and IE were further evaluated in human gastric epithelial cells (GES-1), showing no impact on cell viability up to 200 µg/mL. Figure 3 presents the obtained results.

### 2.5. Antioxidant Capacity: Cell-Free and In Vitro Assay

The antioxidant capacity of HE, WE, and IE was evaluated using two assays: a cell-free assay to assess radical scavenging activity via the DPPH assay, as described in Section 3.9, and an in vitro cellular assay to investigate the promotion of cellular antioxidant responses through the activation of the Nuclear factor erythroid 2-related factor (Nrf2) pathway, a key regulator of oxidative stress, using the in vitro model described in Section 3.10. Table 4 reports the results of the radical scavenging activity.

The DPPH assay is a commonly used method for evaluating the radical scavenging capacity of pure compounds or mixtures by assessing their activity against the 2,2-diphenyl-1-picrylhydrazyl (DPPH) radical [30].

The results are expressed as the 50% radical inhibition capacity (IC_50_), with ascorbic acid selected as the reference compound [31]. The HE sample exhibits an antioxidant capacity comparable to that of ascorbic acid, attributable to its high polyphenol content. In contrast, the WE sample shows the lowest antioxidant capacity, while in the IE sample, this capacity increases but remains 13 times lower than that of the HE sample. These results highlight that, in terms of radical scavenging activity in the scaled-up product, both the solvent used in the extraction method and the extraction technique itself have significant impacts.

**Figure 4:**
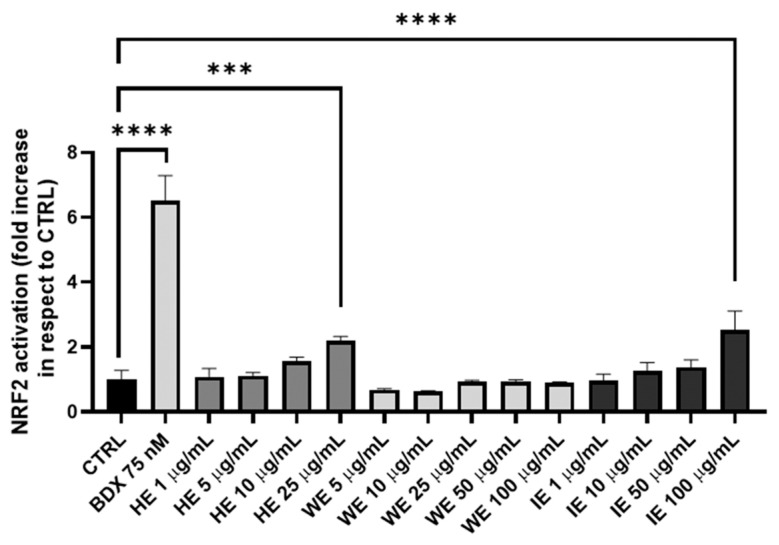
Antioxidant activity of HE, WE, and IE samples in HEK293T cell line with a gene reporter for Nrf2. Bardoxolone (BDX) is the positive control. Data are expressed as mean ± S.E.M. Statistical analysis is performed by one-way ANOVA with Bonferroni’s multiple comparison test (*** *p* < 0.001, **** *p* < 0.0001).

Notably, the green industrial extraction method resulted in a product with twice the radical scavenging activity compared to the laboratory-scale method. Furthermore, the efficacy of HE, WE, and IE in promoting cellular antioxidant responses through the activation of the Nrf2 pathway is reported in Figure 4, which illustrates the dose-dependent response of the tested extracts after 18 h of incubation within the non-toxic concentration range determined via the MTT assay.

Nrf2 activation is significant for the HE and IE at doses of 25 µg/mL and 100 µg/mL, respectively. This response is attributed to the polyphenol content in these extracts. In fact, HE contains 51.612% total polyphenols, representing approximately 50% of its weight. Polyphenols, due to their highly hydroxylated structure, are well-established activators of the Nrf2 pathway through the canonical route. Similarly, the IE sample, containing 2.470% polyphenols (HPLC-UV/DAD method) and approximately 5% of other oxidizing species (Folin–Ciocalteu method), also contains a significant amount of phytocompounds capable of activating this pathway. Conversely, the WE sample, which is lower in both polyphenols and oxidizing molecules, does not exhibit significant Nrf2 activation. These findings confirm that Nrf2 activation correlates directly with the polyphenol and oxidizing molecule content of the extracts [32,33].

### 2.6. In Vitro Studies: Anti-Inflammatory Activity

**Figure 5:**
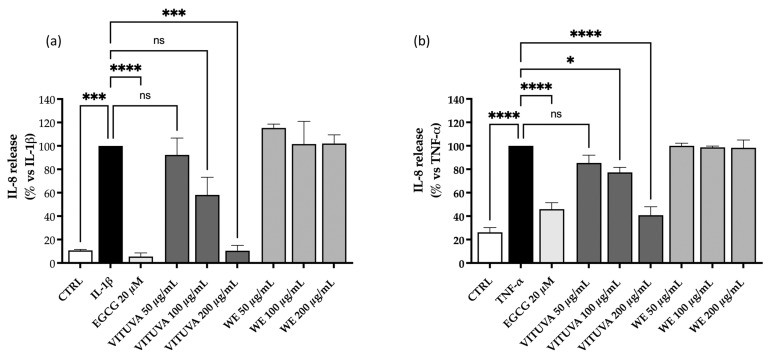
Effect of the WE and IE on the release of IL-8. The activity of the WE and IE is compared in GES-1 cells stimulated by IL-1β (**a**) or TNF-α (**b**). Calculated IC_50_ values of the IE were 101.5 µg/mL and 123.1 µg/mL, respectively. Data are expressed as mean ± S.E.M. Statistical analysis was calculated by one-way ANOVA with Bonferroni’s multiple comparison test (* *p* < 0.05, *** *p* < 0.001, **** *p* < 0.0001 vs. stimulus, ns = not significant).

The anti-inflammatory activity of the extracts WE and IE is evaluated as described in Section 3.10, within the non-toxic concentration range determined via the MTT assay. The IE extract shows inhibitory activity on IL-8 release, with IC_50_ values of 101.5 µg/mL and 123.1 µg/mL against interleukin 1β (IL-1β) and tumor necrosis factor alpha (TNF-α), respectively (Figure 5).

**Figure 6:**
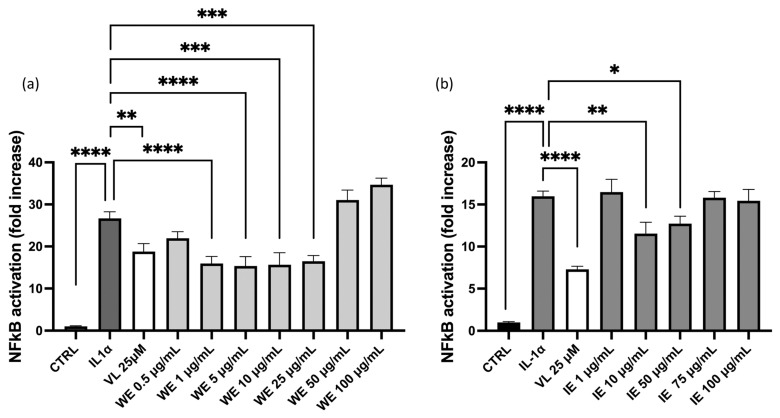
Effect of WE and IE on the NF-κB driven transcription. The activity of WE (**a**) and IE (**b**) is compared in HEK293Tstimulated by IL-1α. Data are expressed as mean ± S.E.M. Statistical analysis was calculated by one-way ANOVA with Bonferroni’s multiple comparison test (* *p* < 0.05, ** *p* < 0.01, *** *p* < 0.001, **** *p* < 0.0001).

Since the nuclear factor kappa-B (NF-κB) is involved in the production of IL-8 and represents a key inflammatory factor, additional experiments were conducted to address this mechanism of action. No concentration–response effect on NF-κB is observed in HEK293T cell line (Figure 6), while no inhibitory activity is observed in GES-1 cells at the concentration tested (Supplementary Data, Figure S2).

For both WE and IE, a slight inhibitory effect is observed in HEK293T-Nrf2 at lower concentrations, while at higher concentrations the effect of these extracts is reversed.

**Figure 7:**
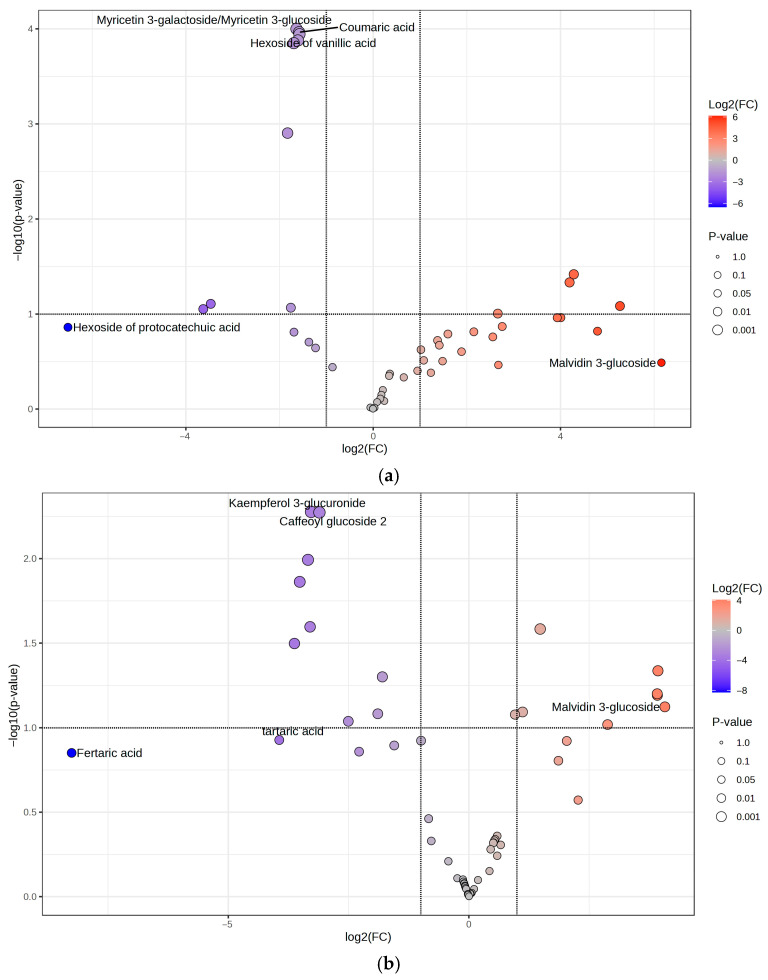
Volcano plots are generated using MetaboAnalyst to explore the phytocompounds in IE and their correlation with the mechanism of action. (**a**) Volcano plot comparing IEs and WEs; in red, the polyphenolic metabolites are more abundant in IE, and in blue, those more abundant in WE. (**b**) Volcano plot comparing HE and IEs; in red, the polyphenolic metabolites are more abundant in HE, and in blue, those are more abundant in IEs.

This observation may reflect the phenomenon of hormesis, an adaptive biological mechanism that enables cells, tissues, and organisms to cope with stress. Hormesis typically shows a biphasic dose–response, characterized by stimulation at low doses and inhibition at higher doses [34]. These data exclude the implication of NF-κB in the inhibitory effect of IL-8 release. On the contrary, the biological effect parallels data obtained in experiments concerning Nrf2-driven transcription, thus suggesting its involvement in the anti-inflammatory mechanism. Nrf2 signaling is mediated not only by IE but also by HE (Figure 4). To identify the phytochemicals linked to this mechanism of action, and considering that these compounds are absent in WE, a volcano plot is generated using MetaboAnalyst to compare IE in respect to WEs and HE. Results are shown in Figure 7.

When comparing the IE to the WE (Figure 7a), it is evident that the two extracts share a limited number of phytocompounds with comparable relative abundance (*n* = 14). However, a higher number of compounds are more abundant in the IE (*n* = 20) compared to those more abundant in the WE (*n* = 13). Specifically, the IE is distinguished from the WE by a higher content of phytocompounds such as procyanidin B and C, laricitrin 3-gluco/galactoside, myricetin 3-glucuronide, quercetin 3-glucuronide, and catechin, all of which are characterized by the presence of an ortho-diphenolic moiety within their structure.

When comparing the IE with the HE, the same compounds are present, with no significant differences between the two extracts (Figure 7b). Indeed, to understand the potential mechanism of action underlying the IE and HE, the focus is not on the significantly different molecules but rather on those similarly extracted in both hydroalcoholic and industrial approaches. In addition to the aforementioned compounds, molecules such as delphinidin 3-gluco/galactoside, petunidin 3-gluco/galactoside, myricetin, myricetin 3-rhamnoside, galloyl glucose, and gallocatechin/epigallocatechin are part of the 54.76% of shared metabolites between the IE and HE that contain an ortho-diphenolic moiety within their structure. This highlights that a significant proportion of the common compounds between the IE and HE are characterized by the presence of the ortho-diphenolic group.

It is well-established in the scientific community that the antioxidant activity of polyphenols is mediated by the upregulation of antioxidant enzymes through Nrf2 activation, a transcription factor associated with antioxidant enzymes and playing a key role in redox homeostasis in cells [35,36]. The activation of Nrf2 is primarily triggered by polyphenolic compounds containing an ortho-diphenol group, which is oxidized to the corresponding quinone. This quinone, being an electrophilic compound, reacts with the thiol groups of KEAP1, thus releasing Nrf2, which activates the transcription of ARE genes. This mechanism of action has been evaluated not only for the polyphenols themselves but also for their microbiota-mediated metabolites, which are active as well and have recently been shown to be safe and free from side effects [37,38,39]. This hypothesis highlights the commonalities between the metabolites extracted from both the HE and IE but not from the WE, explaining why the industrial aqueous extract is the most promising, both from an eco-sustainability and scalability perspective, as well as in terms of pharmacological effects and bioactivity.

Combining the results obtained from chemical-analytical characterization, bioactivity assays on cellular models, and statistical evaluations, the comparison between the aqueous extract obtained at laboratory scale (WE) and the one obtained at industrial scale (IE) highlights the crucial role of the preparation method in developing a pharma/nutraceutical ingredient of interest. The quality and careful manipulation of the starting raw material, in this case, WGSs, represent an essential aspect. However, the preparation technique plays a crucial role in defining the final product’s value.

Although the extraction solvent used for both WE and IE is the same, the superior performance of the industrial extract in terms of bioactivity, phytochemical profile, and environmental sustainability is strictly dependent on the application of an advanced and cutting-edge preparative technology. Specifically, the IE is prepared using an advanced multi-step process designed to maximize the yield and purity of bioactive compounds. The initial hot water infusion, optimized for temperature and mass/volume ratio, facilitates the selective extraction of target molecules.

Subsequent microfiltration and adsorption chromatography ensure the removal of impurities and compliance with safety standards. Ultrafiltration and nanofiltration concentrated the bioactive fraction while preserving its chemical stability. The final spray-drying step, conducted under carefully controlled conditions, produces a highly stable dry powder [29]. This sophisticated technique enhances the product’s quality and efficacy, making it suitable for pharmaceutical applications.

For these reasons, while the preparation of WE using water as the extraction solvent represents an important step in the scale-up and development of IE, it is not sufficient to obtain a product of pharma/nutraceutical interest without the application of advanced industrial technology.

### 2.7. Translational Relevance and Future Perspectives

Although the present study is based on in vitro investigations, its findings can be placed within a broader translational context. Previous studies have already demonstrated the potential of grape by-product derivatives (e.g., seed and pomace) as valuable sources of antioxidant and anti-inflammatory compounds with applications ranging from cosmetics to nutraceuticals and therapeutics.

For example, Salem et al. reported that exhausted grape seed residues can be effectively repurposed into biocompatible cosmetic scrubs that release antioxidant molecules during exfoliation, thereby contributing to zero-waste and circular economy approaches [40]. In a complementary way, Miller et al. showed that dietary supplementation with whole grape powder significantly modulates basal NF-κB signaling and lowers circulating cytokines in vivo, highlighting the systemic anti-inflammatory relevance of grape-derived polyphenols [41]. Similarly, comprehensive analyses have indicated that grape pomace polyphenols may serve as a promising alternative to conventional nonsteroidal anti-inflammatory drugs (NSAIDs) by targeting oxidative stress and inflammatory pathways, supported by both in vitro and in vivo data [42]. Moreover, Magrone et al. recently emphasized that red grape polyphenols exert broad immunomodulatory and metabolic effects, also through interactions with the gut microbiota, while stressing the importance of safety and bioavailability assessments before translation to clinical or cosmetic use [43]. In this perspective, the present study primarily focuses on the valorization of the grappa production chain by-products through a sustainable and green process, scalable at the industrial level, to generate an eco-friendly product. The in vitro investigations are conducted to provide initial insights into its potential applications, in line with and supported by previous evidence reported by other studies, thus confirming the relevance of such models as an essential starting point for future translational developments.

## 3. Materials and Methods

### 3.1. Chemicals

The industrial extract used in this study (proprietary brand denomination Vituva^®^; named with the acronymous IE within the text) was prepared by Distillerie Bonollo Umberto S.p.A. (Mestrino, Italy) using selected white grape skins obtained from Northeast Italian wineries and an extractive food-related procedure based on the sequential combination of an aqueous-infusion and tangential-flow filtration with membranes with varying degrees of selective porosity [29]. 6-Hydroxy-2,5,7,8-tetramethyl-3,4-dihydrochromene-2-carboxylic acid (trolox), Folin–Ciocalteu reagent, sodium carbonate, gallic acid, (+)-catechin, vanillin, hydrochloric acid, potassium chloride, sodium acetate, DMSO, 3-(4,5-dimethyl-2-thiazolyl)-2,5-diphenyl-2*H*-tetrazolium bromide (MTT), IL-1β, ethanol, formic acid and LC-MS grade solvents were purchased from Merck KGaA, Darmstadt, Germany. LC-grade H_2_O (18 MΩ∙cm) is prepared with a Milli-Q H_2_O purification system (Millipore, Bedford, MA, USA). Sepabeads SP207 resin is from Thermo Fisher Scientific (San Jose, CA, USA).

### 3.2. Extracts Preparation

Dried white grape skins derived from controlled and selected wineries in northern Italy were ground in liquid nitrogen before the extraction. The extraction was performed using three different techniques: two at the laboratory scale and one at the industrial scale, namely (i) hydroalcoholic extraction followed by polyphenols enrichment, (ii) water extraction, and (iii) industrial-scale green extraction.

For the hydroalcoholic extract (HE), raw material is added in a H_2_O/EtOH/formic acid mixture (70:30:0.1; % *v*/*v*) with a biomass-volume ratio of 1:20 (*w*/*v*) for 18 h under magnetic stirring at room temperature. The resulting extract is centrifuged, filtered, and dried using a rotary evaporator. For polyphenols enrichment, a specific resin (Sepabeads SP207, Thermo Fisher Scientific, San Jose, CA, USA) is activated for 48 h in EtOH and under magnetic stirring and packed into a flash chromatography column (diameter 4 cm, length 25 cm). The extract is resuspended in water (250 mL), loaded onto the resin, and washed with water until complete elimination of sugars (control with burnt TLC). The enriched extract is then eluted with EtOH/formic acid 0.01% and dried by rotary evaporation.

For the water extract (WE), H_2_O is added to the raw material with a biomass–volume ratio of 1:20 (*w*/*v*) at 100 °C for one hour under magnetic stirring, followed by 23 h at room temperature under magnetic stirring. The obtained extract is centrifuged (5000× *g* for 15 min), and the collected supernatant is dried by rotary evaporation.

The conditions used combine the aim of achieving both complete extraction and enrichment of grape polyphenols with the development of a low-cost and eco-sustainable method that can be scaled up in an industrial facility. Moreover, water extraction of polyphenols is well documented, and a limited heating period (1 h) does not degrade polyphenols [44].

For the industrial-scale green extraction (IE), white grape skins (not ground) were processed using the water-based extraction method detailed in Section 3.2.

### 3.3. HPLC-HRMS Analysis

The HE sample is resuspended (10 mg/mL) in CH_3_OH/H_2_O (50:50, % *v*/*v*) and diluted for the analysis 1:2 in H_2_O/HCOOH, 100/0.1% *v*/*v* (mobile phase A) to obtain the final concentration of 5 mg/mL. The WE sample is prepared in H_2_O + FA 0.1% (with FA for formic acid) at a final concentration of 5 mg/mL. The IE sample was prepared in H_2_O+ FA 0.1% at a final concentration of 2 mg/mL. All extract samples are spiked with the internal standard (6-hydroxy-2,5,7,8-tetramethyl-3,4-dihydrochromene-2-carboxylic acid, trolox) at the final concentration of 1 × 10^−4^ M.

The analyses are performed in triplicate by liquid chromatography high-resolution mass spectrometry (LC-HRMS). Compounds were separated on a reversed-phase Agilent Zorbax SB-C18 column (150 × 2.1 mm, i.d. 3.5 µm, CPS analitica, Milan, Italy) by using a multi-step gradient of mobile phase A H_2_O- HCOOH (100:0.1, % *v*/*v*) and phase B CH_3_CN- HCOOH (100:0.1, % *v*/*v*). Detection was carried out with an LTQ Orbitrap XL mass spectrometer (Thermo Fisher Scientific, San Jose, CA, USA), as described by Della Vedova et al. [33]. The spectra were acquired in both negative and positive ion modes. Xcalibur 4.0 and Chromeleon Xpress 6.80 (Thermo Fisher Scientific, San Jose, CA, USA) were used for instrument control.

### 3.4. Data Processing

#### 3.4.1. Targeted Annotation

Xcalibur 4.0 was used for spectra analysis. Data were evaluated by both targeted and untargeted approaches. The targeted approach was based on a database of compounds of *Vitis Vinifera* L. built in our laboratory [35], and annotated spectra were confirmed using the free online tools HMDB 5.0 and CFM-ID 4.0. According to the Metabolomics Standards Initiative (MSI) guidelines, the annotation level of the putative identities assigned to the data in the targeted approach was classified as MSI level 2; the compound annotation was carried out by using the exact mass (5 ppm of mass tolerance), the isotopic and fragmentation patterns. The untargeted approach is based on GNPS Library Search and GNPS Molecular Networking free online tools, with an annotation level MSI level 3 [45].

#### 3.4.2. Spectral Organization Through Molecular Networking with GNPS

For the classical molecular networking workflow, a molecular network was created using the online (https://ccms-ucsd.github.io/GNPSDocumentation/, accessed on 18 June 2024) on the GNPS website (http://gnps.ucsd.edu, accessed on 18 June 2024). The data are filtered by removing all MS/MS fragment ions within +/− 17 Da of the precursor m/z. MS/MS spectra are window-filtered by choosing only the top 6 fragment ions in the +/− 50 Da window throughout the spectrum. The precursor ion mass tolerance is set to 2.0 Da, and a MS/MS fragment ion tolerance is set to 0.5 Da. A network is then created where edges are filtered to have a cosine score above 0.7 and more than 4 matched peaks. Furthermore, edges between two nodes exist only if each of the nodes appears in the other’s top 10 most similar nodes.

Finally, the maximum size of a molecular family is set to 100, and the lowest-scoring edges are removed from molecular families until the molecular family size falls below this threshold. The spectra in the network are searched against GNPS spectral libraries, which are filtered in the same manner as the input data. All matches kept between network spectra and library spectra are retained only if they have a cosine score above 0.7 and at least 4 matched peaks [46].

### 3.5. Statistical Analysis

Statistical analysis was performed using MetaboAnalyst 6.0, a free online tool useful for complex bioinformatics and statistical needs for metabolomics data, including raw spectra processing, functional analysis, and integration [47]. The resulting LC-HRMS metabolomics data were analyzed using a sparse variant PLS data analysis (sPLS-DA), which combines most predictive and discriminant features selection and classification in a one-step procedure. This statistical approach is also applied for the geographical characterization of secondary metabolites of plants and biological features [48,49]. For all metabolites identified in HE, WE, and IE samples, the relative abundance was calculated as a ratio between the area under the curve (AUC) of the considered compound and the AUC of the internal standard (trolox, 100 µM). A matrix containing the relative concentration of all metabolites is uploaded to MetaboAnalyst as a CSV file. The samples were organized in columns as unpaired data, normalized by their median, and scaled by centering the mean value. For the volcano plot comparing HE and IE, a separate matrix containing only the two compared extracts was created as a CSV file and uploaded to MetaboAnalyst, using the same normalization parameters.

Statistical analysis of cell experiments, including IC_50_ calculation, was carried out on three independent biological replicates using a one-way ANOVA test, followed by Bonferroni post hoc analysis in Prism 9.0 software (GraphPad Software Inc., San Diego, CA, USA). Data are expressed as mean ± S.E.M., relative to the pro-inflammatory stimulation, which was arbitrarily assigned the value of 100%.

### 3.6. Total Polyphenol Content

Folin–Ciocalteu and HPLC-UV/DAD methods were applied to evaluate the total polyphenol content in HE, WEs, and IEs. The total polyphenol content is determined by the Folin–Ciocalteu colorimetric method, as reported by Baron et al. [44], using catechin as an external calibration standard in a concentration range from 1 to 1000 µg/mL, and by HPLC-UV/DAD, as reported by Della Vedova et al. [33] with minor modifications. Specifically, HE, WE, and IE samples solutions were prepared and diluted in H_2_O/HCOOH, 100/0.1, % *v*/*v* (mobile phase A) to a final concentration of 2 mg/mL. An aliquot of 25 µL of each sample was analyzed in triplicate using a high-performance liquid chromatography (HPLC) system (Ultimate3000, Thermo Fisher Scientific, Milan, Italy) equipped with a diode array detector (DAD) as analyzer and an Agilent Zorbax SB-C18 reverse phase column (150 × 2.1 mm, i.d. 3.5 μm, CPS analitica, Milan, Italy).

The same eighty-minute multistep gradient is used for the separation of the polyphenols present in all three extracts. The flow rate is set at 250 µL/min, the column is maintained at 40 °C, and the DAD is monitored at 285 nm. For quantification, a calibration curve with catechin as an external standard is built in the concentration range from 0.1 to 50 µg/mL. Each chromatographic peak is integrated, and the AUC is interpolated into the calibration curve. The total amount of polyphenols is calculated by the sum of the concentration of each identified polyphenol, and the results are reported as a % *w*/*w*.

### 3.7. Tannin Content

Vanillin assay [50] was carried out for the determination of total tannin content in HE, WE, and IE samples. Briefly, 2.5 µL of each sample (initial concentration 10 mg/mL) was tested in a 96-well plate, adding 150 µL vanillin solution (4% solution in CH3OH) and 75 µL of HCl 37%. A calibration curve was built using (+)-catechin (concentration range 10–1000 µg/mL) with the same protocol as the samples. The mixtures were incubated at room temperature for 15 min, and the absorbance was measured at 500 nm against the blank using a microplate reader (BioTek’s PowerWave HT, Winooski, VT, USA).

The results are expressed as % *w*/*w* (grams of polyphenols per one gram of starting material or dry extract).

### 3.8. Anthocyanin Content

Total anthocyanin content was determined by UV-Vis spectroscopy according to the method described by Giusti et al. [51]. Quantification was performed in triplicate. A total of 8 mg/mL stock solution for WE sample, 1 mg/mL for HE sample, and 5 mg/mL for IE are prepared and diluted with pH 1 buffer (0.025 M potassium chloride solution at pH 1) and pH 4.5 buffer (0.4 M sodium acetate solution at pH 4.5) to obtain a final concentration of 1 mg/mL, 50 µg/mL and for the WE, HE and IE, respectively. The absorbance of each sample was measured at 520 nm (λ of maximum absorption) and 700 nm using a Shimadzu UV 1900 spectrophotometer (Shimadzu, Milan, Italy). The concentration of anthocyanins in the stock solution is expressed in mg/L and is calculated following Equation (1):

(1) $$Concentrazion\;of\;anthocyanins\;\left(\frac{mg}{L}\right)=\frac{A\;\times \;MW\;\times \;DF\;\times 1000}{\left(\varepsilon \;\times \;1\right)}$$

Equation (1) was used to calculate, in mg/L, the total anthocyanin concentration.

*A* = (A520–A700)pH1 − (A520–A700)pH4.5, *MW* = molecular weight of malvidin glucoside (449.4 g/mol), *DF* = dilution factor, and *ε* = molar absorbance of malvidin glucoside (20,200 L mol^−1^ cm^−1^). The results are expressed as mg of anthocyanins in 100 mg of extract powder (%).

### 3.9. Radical Scavenging Activity

The radical scavenging activity was evaluated using the 2,2-diphenyl-1-picrylhydrazyl (DPPH) assay, as reported by Della Vedova et al. [35]. Specifically, an aqueous solution of WE was diluted to obtain final extract concentrations in the range of 0.1 to 0.5 mg/mL, the HE was diluted in EtOH/H_2_O (50:50, % *v*/*v*) to obtain final extract concentrations in the range of 0.1 to 10 µg/mL, and IE extract was diluted in EtOH/H_2_O (50:50, % *v*/*v*) to obtain final extract concentrations in the range of 10 to 100 µg/mL. A total of 1 mL of acetate buffer (100 mM, pH 5.5) and 1 mL of ethanol were added. To each 500 µL aliquot of diluted extract, 1 mL of acetate buffer (100 mM, pH 5.5) and 1 mL of ethanol were added. Finally, 500 µL of an ethanolic solution of DPPH (500 µM) was added, and the samples were incubated in the dark for 90 min. The absorbance was measured at 517 nm using a Shimadzu UV-1900 spectrophotometer (Shimadzu, Milan, Italy). The percentage of inhibition was calculated using Equation (2), and results are expressed as mean ± SD. IC_50_ values obtained were compared with those of trolox and ascorbic acid, molecules that are known to possess strong radical scavenging activity.

I % = (Abs (blank sample) − Abs (sample))/(Abs (blank sample)) × 100(2)

Equation (2) was used to calculate the percentage of inhibition of the DPPH radical.

### 3.10. In Vitro Assays

#### 3.10.1. Cell Lines and MTT Assay

The cell viability for all the concentrations tested in the antioxidant and anti-inflammatory assay was verified by MTT assay on stable labeled HEK293T-NRF2 and HEK293T-NF-Κb cells. Reagents used for the maintenance of both HEK293 cell lines (DMEM low glucose, heat-inactivated FBS, penicillin, and streptomycin) were provided by Euroclone (Euroclone S.p.A., Pero-Milan, Italy). GES-1 cells, derived from normal human gastric epithelium, were kindly provided by Dr. Dawit Kidane-Mulat (Howard University, College of Medicine, Washington, DC, USA). The reagents used for cell culture (RPMI 1640 medium, trypsin-EDTA, antibiotics, and amino acids) were provided by Gibco (Life Technologies Italia, Monza, Italy), while fetal bovine serum (FBS) and disposable material were provided by Euroclone (Euroclone S.p.A., Pero-Milan, Italy).

After 18 h incubation with bardoxolone (75 nM), DMSO 4%, and the samples (HE from 1 to 250 µg/mL; WE from 1 to 250 µg/mL; IE from 1 to 250 µg/mL), 11 µL 5 mg/mL MTT reagent was added for 5 h. After medium removal, cells were lysed, and MTT was solubilized by adding 100 µL of DMSO. The 96-well plate was shaken for 15 min, and the absorbance was measured at 575 nm using a plate reader (BioTek PowerWave HT, Winooski, VT, USA). Cells incubated with DMSO (<0.1%) were used as a control for 100% cell proliferation.

#### 3.10.2. Antioxidant Activity

The Nrf2/ARE Responsive Luciferase HEK293 cell line was used for the evaluation of antioxidant activity. The cells were seeded in white 96-well plates BRANDplates^®^, cell grade (BRAND GMBH + CO. KG, Wertheim, Germany) with a density of 10,000 cells/well. The cells were pretreated with different concentrations of WE (1–250 µg/mL) and IE (1–100 µg/mL) extracts for 18 h in complete medium (DMEM, 10% FBS, 1% L-glutamine, 1% penicillin/streptomycin), and bardoxolone (BDX) was used as a reference compound. To avoid reading interference, the medium was removed and 50 µL of PBS was added to each well. A total of 50 µL of ONE-Glo™ Luciferase Assay Substrate (purchased from Promega Corporation, Madison, WI, USA) was added directly to the cells, and luciferase activity was measured using a luminometer (Synergy 2, BioTek^®^ Instruments, Inc., Highland Park, VT, USA). Experiments were performed with technical and biological replicates.

#### 3.10.3. Anti-Inflammatory Activity

The anti-inflammatory activity of extracts is evaluated using the NF-κB reporter (Luc) HEK293 cell line purchased from BPS Bioscience (San Diego, CA, USA). Briefly, the HEK293 NF-κB cell line was seeded in a 96-well blank plate (BRANDplates^®^, cell grade) at a density of 15,000 cells/well. After 6 h, cells were pretreated with different concentrations of WE (0.5–250 µg/mL) and IE (1–100 µg/mL) extracts for 18 h in complete medium (MEM, 10% FBS, 1% NEAA, 1 mM Na pyruvate, 1% penicillin/streptomycin) and 5-(3′,4′-dihydroxyphenyl)-γ-valerolactone (VL) was used as reference compound [38]. An inflammatory state was induced for 6 h with 0.01 ng/mL IL-1α.

After incubation, the medium was removed and replaced with 50 µL MEM per well. Aliquots of 50 µL of ONE-Glo™ Luciferase Assay Substrate (Promega Corporation, Madison, WI, USA) were added directly to the cells, and luciferase activity was measured using a luminometer (Synergy 2, BioTek^®^ Instruments, Inc., Highland Park, VT, USA). Experiments are performed with both technical and biological replicates.

NF-κB-driven transcription was also measured in GES-1 cells by transient transfection with 50 ng/well NF-κBLuc plasmid (kindly provided by Dr. N. Marx, Department of Internal Medicine-Cardiology, University of Ulm, Ulm, Germany). In brief, GES-1 cells were seeded in 24-well plates (3 × 10^5^ cells/well) for 48 h, then the medium was replaced with FBS-free medium, and transfection was performed using Lipofectamine^®^ (Invitrogen™, Thermo Fisher Scientific, Waltham, MA, USA)according to the manufacturer’s instructions and previous reports [52]. On the following day, transfected cells are treated with pro-inflammatory cytokines (IL-1β or TNF-α, 10 ng/mL) and increasing concentrations of plant extracts for 6 h. Luciferase activity was measured using a luminometer (Victor™ X3, PerkinElmer, Waltham, MA, USA) after the addition of Britelite™ Plus reagent (PerkinElmer, Waltham, MA, USA).

IL-8 release was measured by ELISA assay using the Human IL-8 ABTS Standard ELISA Development Kit (Peprotech, London, UK) as previously described [52]. Briefly, EIA/RIA clear plates (Merck, Darmstadt, Germany) were coated with a primary anti-IL-8 antibody. The following day, 100 μL of cell culture medium was added for 2 h, then the plates were washed with PBS 1X and incubated with a biotinylated secondary antibody. An HRP–avidin–biotin complex was formed to mediate the colorimetric reaction. The absorbance was measured at 405 nm using a photometer (Victor™ X3, PerkinElmer, Waltham, MA, USA) and compared with a calibration curve constructed with standard human IL-8 (0–1000 pg/mL).

## 4. Conclusions

In this work, it is demonstrated that the industrial scale-up product IE (Vituva^®^) is positioned, from a qualitative and semi-quantitative point of view, between the extract with a more varied polyphenolic profile (HE) and the one predominantly containing phenolic acids (WE). For IE, a tangential flow extraction facility enables the extraction of less polar compounds, such as anthocyanins, which are detected in HE but not in WE, for which a simpler extraction method was employed on the laboratory scale.

The HE is not considered for industrial scale-up due to the use of ethanol as a solvent and its lower cell viability. Despite its richer polyphenol profile, HE proves more toxic compared to WE and IE. This higher abundance of polyphenol in HE, rather than representing an advantage, constitutes a disadvantage because of its increased toxicity. This phenomenon is explained by the concept of hormesis, which suggests that excessive concentrations of polyphenols may exceed organism-specific thresholds, becoming toxic rather than beneficial [34].

Additionally, this study demonstrates that WGSs represent a valuable source of polyphenols, extractable through an eco-sustainable method. The industrial scale-up leads to the production of IE, which not only has a richer polyphenol profile but also exhibits both anti-inflammatory and antioxidant activity, highlighting the importance of the circular economy and the valorization of natural by-products. Advanced analytical methodologies, including chemoinformatics and statistical analysis, are crucial in this study for evaluating the bioactivity and determining the most effective extraction method. These techniques, combined with in vitro biological assays, allow for a comprehensive evaluation of the extracts’ activity and provide insights into the impact of different extraction methodologies on the final bioactive profile. Specifically, these methods also highlight the common features between IE and HE and how their high similarity in terms of phenolic metabolites, characterized by the presence of the ortho-diphenol moiety, has a significant impact on the anti-inflammatory and antioxidant effects of both. Moreover, the results clearly indicate that the advanced industrial preparation method adopted for IE plays a central role in obtaining an extract with enhanced bioactivity and stability. Despite using the same extraction solvent as WE, the combination of tangential flow filtration, selective adsorption, and spray drying processes enables the selective enrichment of polyphenolic compounds, while preserving their biological activity. This demonstrates how the technological approach is not merely a means of scale-up but a critical factor in the development of a high-quality product for pharma/nutraceutical applications.

In vitro studies further support the biological relevance of IE. The MTT assay reveals that both IE and WE are non-toxic at concentrations up to 100 µg/mL, in contrast to HE, which showed toxicity at concentrations as low as 50 µg/mL. Interestingly, results show that WE exhibits a strong anti-inflammatory effect starting from 1 µg/mL, which is not associated with Nrf2 activation, as WE does not modulate Nrf2 activity in human embryonic kidney (HEK) 293 cells. On the other hand, IE not only demonstrates potent anti-inflammatory activity (from 1 µg/mL) but also significantly modulates the Nrf2 pathway at higher concentrations (100 µg/mL). Additionally, IE effectively reduces interleukin-8 (IL-8) release in GES-1 cells, namely a key pro-inflammatory cytokine in gastritis, further supporting the biological relevance of this industrially scaled-up extract. Furthermore, IE significantly activates the Nrf2 pathway, a key regulator of oxidative stress, confirming its potential antioxidant properties.

The anti-inflammatory effects of IE are also evident, as it inhibited IL-8 release and showed substantial inhibition of pro-inflammatory cytokines, IL-1β and TNF-α, in GES-1 cells. These findings suggest that the biological effects observed are directly correlated with the polyphenol and oxidizing molecule content of the extracts. Specifically, considering the results obtained from the GES-1 cell line, IE shows potential as a nutraceutical product, particularly for the reduction in gastric inflammation. Overall, the phytochemical analysis performed by LC-HRMS, combined with cheminformatics methodologies and the evaluation of biological activity in in vitro models, highlights that IE represents an eco-sustainable extract derived from agri-food waste materials like WGSs, exhibiting anti-inflammatory activity mediated by Nrf2 signaling due to its significant content of phenolic compounds bearing the ortho-diphenol moiety. Future studies will focus on the development of a finalized nutraceutical product incorporating IE, aiming at further elucidating its mechanism of action and clinical relevance.

## Figures and Tables

**Table 1:** Yield of extraction of hydroalcoholic extract (HE), water-based extract (WE), and industrial-scaled extract (IE) samples.

Sample	Yield of Extraction (%)
Hydroalcoholic extract (HE)	9.27%
Water-based extract (WE)	24%
Industrial-scaled extract (IE)	20%

**Table 2:** List of all the metabolites putatively identified with a targeted approach in HE, WE, and IE samples by the HPLC-HRMS approach.

Name	[M-H]^−^	[M]^+^/[M+H]^+^	RT	MS^2^ (Negative Ion Mode)	MS^2^ (Positive Ion Mode)	HE	WE	IE
Caffeic acid	179.03443		2.32	135		x	x	nd
Caffeoyl glucoside	341.08725		3.17	179-251-281		x	x	x
Caffeoyl glucoside 1	341.08732		3.37	179-221-251-281		nd	x	x
Caffeoyl glucoside 2	341.08722		3.97	179-221-251-281		nd	x	x
Caftaric acid	311.04031		2.88	149-179		x	x	nd
Catechin	289.07121	291.08685	3.43	245-205-203-247-179	123-139-151-165	x	x	x
Catechin gallate/epicatechin gallate	441.08217	443.09781	14.43	169-289	139-151-273-291	x	x	nd
citric acid	191.0191772		1.78	191		nd	nd	x
Coumaric acid	163.03952		4.57	119		nd	x	nd
Coutaric acid	295.04539		4.47	175		x	x	x
Dihydrokaempferol-3-rhamnoside	433.11347		32.21	225-269-359-387		nd	x	nd
Dihydroquercetin-3-acetylglucoside	507.11386		30.57	329-344-345		nd	x	nd
Dihydroquercetin-3-rhamnoside	449.10838		20.13	151-285-302-323-403		nd	x	nd
Epicatechin	289.07121	291.08685	5.64	245-205-203-247-179	123-139-151-165	x	x	x
Ethyl gallate	197.045		5.80	153		x	x	x
Fertaric acid	325.05595		6.1	163-193-265		x	x	x
Gallic acid	169.0137		1.46	125		x	x	x
Gallocatechin/Epigallocatechin	305.06612		1.8	179-221-219-261		x	x	x
Galloyl glucose	331.06652		1.52	169-271		x	nd	x
Hexoside of protocatechuic acid	315.0716		2.88	153		x	x	nd
Hexoside of vanillic acid	329.08725		6.46	191-299-197		x	x	nd
Isorhamnetin 3-glucoside	477.1033		27.05	314-315		x	nd	nd
Isorhamnetin-rhamnoside	461.10838		6.47	299		x	nd	nd
Kaempferol 3-galactoside/Kaempferol 3-glucoside	447.09273		24.54	255-285		x	nd	x
Kaempferol 3-glucuronide	461.07222		26.99	229-257-285-329-346		nd	nd	x
Kaempferol 3-rutinoside		595.16651	15.77		387	nd	nd	x
Laricitrin 3-galactoside/Laricitrin-3-glucoside	493.09821		20.90	331-317		x	x	x
Laricitrin-3-acetylglucoside		537.12478	8.95		493	nd	nd	x
Malvidin 3-(6″-acetyl)-glucoside		535.14515	21.68	331		x	nd	
Malvidin 3-glucoside/Malvidin 3-galactoside		493.13459	5.99	331		x	nd	x
Malvidin-3-(6″-coumaroyl)-glucoside		639.17137	38.26	331		x	x	x
Myricetin	317.02974		27.80	249		x	nd	nd
Myricetin 3-galactoside/Myricetin 3-glucoside	479.08256	481.0982	11.4	179-317	319	x	x	x
Myricetin 3-glucuronide	493.06183	495.07747	11.74	317	319	x	x	x
Myricetin-3-rhamnoside	463.08758		19.42	301		x	x	nd
p-Coumaric acid	163.03952	165.05516	4.40	119		x	nd	x
p-Coumaroyl-glucose 1	325.09234	327.10798	4.27	265-235-163	147-281-299	x	x	x
p-Coumaroyl-glucose 2	325.09234	327.10798	5.57	265-235-163	147-165-291	x	x	x
Peonidin 3-(6″-coumaroyl)-glucoside		609.1608	37.82	301		x	nd	nd
Peonidin 3-glucoside		463.12403	5.58	301		x	nd	nd
Petunidin 3-glucoside		479.11894	3.46	317		x	nd	nd
p-Hydroxybenzoic acid	137.02387		3.72	108-137		x	nd	nd
Piceid/Resveratrol-glucose	389.12364		13.2	227-320-343-360		x	nd	nd
Procyandin B peak1	577.13459	579.15023	2.38	289-407-425-451	247-291-301-409-427	x	x	x
Procyanidin B peak2	577.13459	579.15023	3.55	289-407-425-451	247-291-301-409-427-453-533	x	x	x
Procyanidin B peak3	577.13459	579.15023	5.25	289-407-425-451	247-287-291-301-409-427	x	x	x
Procyanidin B peak4	577.13459	579.15023	10.24	289-407-425-451	409-427-435-439-453	x	x	x
Procyanidin trimer peak 1	865.19798	867.21362	3.93	696-793	409-577-698-715	x	x	nd
Procyanidin trimer peak 3	865.19798	867.21362	8.88	577	579-715-823	x	x	nd
Protocatechuic acid	153.01878		2.29	109-123		x	x	nd
protocatechuic acid	153.01962		2.93		88-108-113-123-153	nd	nd	x
Quercetin	301.03483		43.75	151-273-301		x	nd	x
Quercetin 3-galactoside/Quercetin 3-glucoside	463.08765	465.10329	17.68	301-271-311	303	x	x	x
Quercetin 3-glucuronide	477.06758	479.08255	18.21	301	303	x	x	x
Quercetin 3-rutinoside	609.14555		18.39	301		x	nd	nd
Quercetin-3-rhamnoside	609.14555		18.39	301		x	nd	x
Quercetin-3-rhamnoside-glucoside	609.14555		18.39	301		x	nd	nd
Syringetin		347.07662	55.14			nd	nd	x
Syringetin 3-(6″-coumaroyl)-glucoside	653.15064		55.49	301-345-386		x	nd	nd
Syringetin 3-glucoside/Syringetin 3-galactoside	507.11386	509.1295	29.61	329-344-345	347	x	x	x
tartaric acid	149.008613		1.29	58-86-103-105-149		nd	nd	x
Vitisin A		561.12443	13.28		399	x	nd	x
Vitisin B		519.15025	14.05		355	x	nd	nd

x means present, nd means not detected.

**Table 3:** Total polyphenol, anthocyanin, and tannin contents in HE, WE, and IE samples.

Assay	HE (Mean ± SD)	WE (Mean ± SD)	IE (Mean ± SD)
Polyphenol content (HPLC-UV/DAD)	43.682% ± 2.665%	2.999% ± 0.052%	2.470% ± 0.059%
Polyphenol content (Folin–Ciocalteu)	51.612% ± 1.674%	2.714% ± 0.098%	7.154% ± 0.412%
Anthocyanin content	1.232% ± 0.060%	<0.005%	0.141% ± 0.034%
Tannin content	13.637% ± 2.233%	2.471% ± 0.079%	2.479% ± 0.057%

Mean ± standard deviation (%).

**Table 4:** Radical scavenging activity of HE, WE, and IE samples compared to ascorbic acid, expressed as mean ± standard deviation.

Assay	HE (Mean ± SD)	WE (Mean ± SD)	IE (Mean ± SD)	Ascorbic Acid (Mean ± SD)
Radical scavenging capacity IC_50_ (µg/mL)	4.388 ± 0.041	113.565 ± 2.148	58.335 ± 7.217	3.918 ± 0.047

## Data Availability

The data presented in this study are available on request from the corresponding author. The data are not publicly available due to ongoing analyses and future planned publications.

## Supplementary Materials

The following supporting information can be downloaded at: https://www.mdpi.com/article/10.3390/ph18091373/s1. Figure S1. Molecular network of WGS extracts analyzed by LC-MS/MS visualized using Cytoscape. Node colors represent the WGS extracts (red for HE in negative ion mode, light blue for HE in positive ion mode, green for WE in negative ion mode, orange for WE in positive ion mode, yellow for IE in negative ion mode and in gray for IE in positive ion mode) and the respective MS2 spectral counts indicating presence and absence of metabolites. Figure S2. Effect of WE and IE on the NF-κB driven transcription. IE was further assessed in GES-1 cells stimulated by IL-1β or TNF-α. Statistical analysis was calculated by one-way ANOVA with Bonferroni’s multiple comparison test (** *p* < 0.01).
