# Supplementary material for: White Grape Skin Extraction, Analytical Profile, and Biological Activity: From the Laboratory to the Industrial Scale Within a Circular Economy Framework
Source: Pharmaceuticals (Basel). 2025 Sep 13;18(9):1373. doi: 10.3390/ph18091373 (PMC12472989; doi:10.3390/ph18091373)
Supplement: Supplementary file 1 [file pharmaceuticals-18-01373-s001.zip › pharmaceuticals-3835293-supplementary.pdf]

Supplementary data

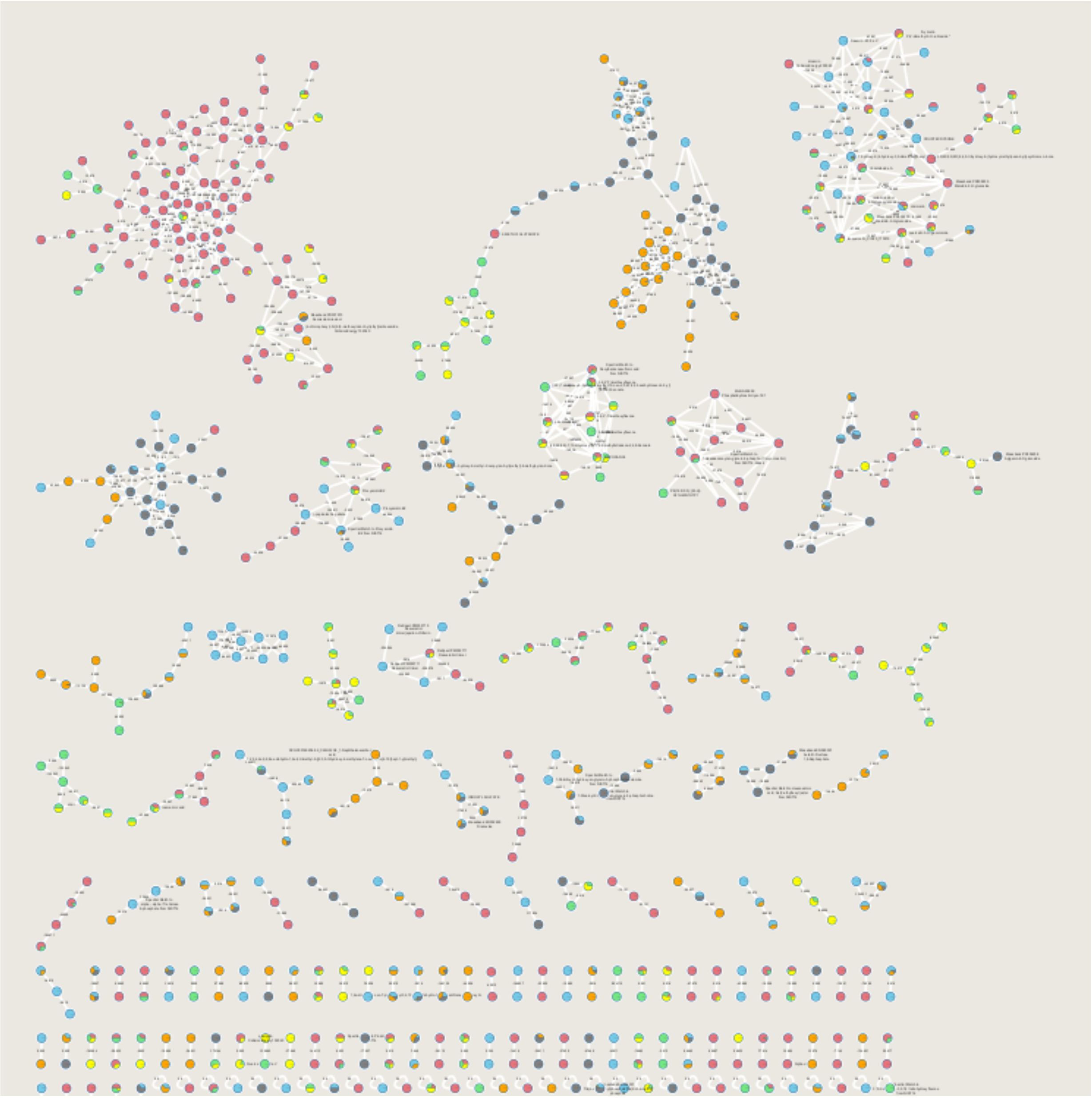

Figure S1. Molecular network of WGS extracts analyzed by LC-MS/MS visualized using Cytoscape. Node colors represent the WGS extracts (red for HE in negative ion mode, light blue for HE in positive ion mode, green for WE in negative ion mode, orange for WE in positive ion mode, yellow for IE in negative ion mode and in gray for IE in positive ion mode) and the respective MS2 spectral counts indicating presence and absence of metabolites.

)

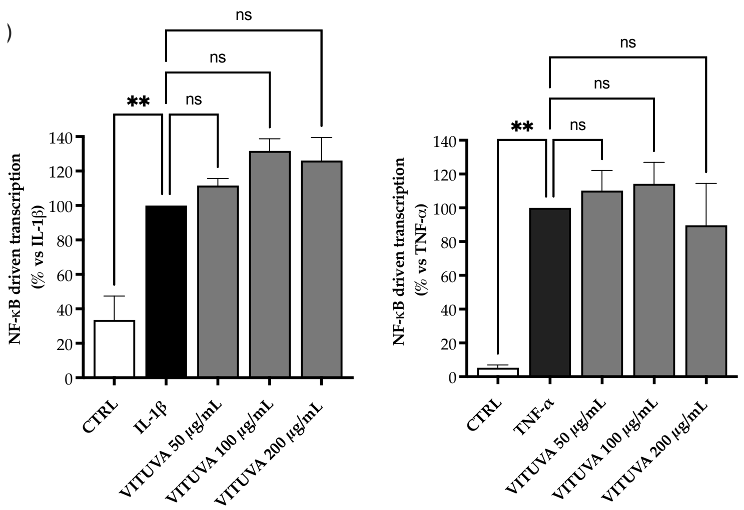

Figure S2. Effect of WE and IE on the NF-κB driven transcription. IE was further assessed in GES-1 cells stimulated by IL-1β or TNF-α. Statistical analysis was calculated by one-way ANOVA with Bonferroni's multiple comparison test (**p < 0.01).
